# A. V. C. Silver Anniversary

**Published:** 1899-06

**Authors:** 


### EDITORIAL.

### A. V. C. SILVER ANNIVERSARY.

THE twenty-fifth anniversary of the establishment of a veterinary college in the United States is an event of no little importance in a profession the youngest of them all, and one that has moved with no uncertain steps to a recognized place among the higher vocations of man.

The record of the American Veterinary College as a factor in higher veterinary education is a grand one, and fittingly adorns an institution that has sent forth so many worthy representatives of the profession. The chief director for many years has earned an international fame, and the school over which he has so worthily presided been accorded a recognition abroad seldom given an American medical school. Her graduates, now exceeding six hundred and more, scattered all over our own land and dotting many parts of foreign soil, have contributed much to the elevation of the profession wherever they have set foot.

Such an occasion should recall every dutiful son of this institution to the home of his alma mater and to join in the festivities of so momentous an occasion in veterinary education in America. There will be old friendships to renew; there will be a mingling of pleasure and gladness for all; a reminiscent period for each one, and a comparison of college days then and now. There will be many an experience to relate, many a disappointment to tell of, many a victory to record, and there will be one more opportunity of being for a day with that grand old man whom every A. V. C. boy loves, whom every member of the American veterinary profession honors, the Dean of the American Veterinary College, Prof. A. Liautard.

